# Interstitial lung disease is not rare in immune-mediated necrotizing myopathy with anti-signal recognition particle antibodies

**DOI:** 10.1186/s12890-021-01802-1

**Published:** 2022-01-10

**Authors:** Yongpeng Ge, Hanbo Yang, Xinyue Xiao, Lin Liang, Xin Lu, Guochun Wang

**Affiliations:** Department of Rheumatology, The Key Laboratory of Myositis, China-Japan Friendship Hospital, Yinghua East Road, Chaoyang District, Beijing, 100029 China

**Keywords:** Immune-mediated necrotizing myopathy, Interstitial lung disease, Anti-signal recognition particle antibodies, Nonspecific interstitial pneumonia

## Abstract

**Objectives:**

The purpose was to clarify the characteristics of interstitial lung disease (ILD) in immune-mediated necrotizing myopathy (IMNM) patients with anti-signal recognition particle (SRP) antibodies.

**Methods:**

Medical records of IMNM patients with anti-SRP antibodies were reviewed retrospectively.

**Results:**

A total of 60 patients were identified. Twenty-seven (45.0%) patients were diagnosed with ILD based on lung imaging: nonspecific interstitial pneumonia (NSIP) in 17 patients (63.0%) and organizing pneumonia in 9 patients (33.3%). Reticulation pattern was identified in 17 patients (63.0%) whereas 10 cases (37.0%) showed ground glass opacity and patchy shadows by high-resolution computed tomography (HRCT). Pulmonary function tests (PFTs) were available in 18 patients, 6 (33.3%) and 10 (55.6%) patients were included in the mild and moderate group, respectively. The average age at the time of ILD onset was significantly older than those without ILD (48.6 ± 14.4 years vs. 41.2 ± 15.4 years, *p* < 0.05), and the frequency of dysphagia in the ILD group was higher than the group without ILD (*p* < 0.05). Long-term follow-up was available on 9 patients. PFTs were stable in 8 (88.9%), and the HRCT remained stable in 6 (66.7%) patients.

**Conclusions:**

ILD is not rare in IMNM patients with anti-SRP antibodies, most being characterized as mild to moderate in severity. NSIP is the principal radiologic pattern, and ILD typically remains stable following treatment.

## Introduction

Idiopathic inflammatory myopathies (IIMs) are a group of clinically heterogeneous, autoimmune inflammatory disorders characterized by muscular weakness and multi-system involvement such as lung and skin. Dermatomyositis (DM), antisynthetase syndrome (ASS) and inclusion body myositis (IBM) have long been recognized as distinct subtypes of IIMs. However, in recent years, it has been recognized that muscle biopsies from some patients with myositis have significant myofiber necrosis yet minimal, if any, lymphocytic infiltrates. These patients are now widely recognized to have immune-mediated necrotizing myopathy (IMNM).

The presence of myositis specific autoantibodies (MSAs) and myositis associated autoantibodies (MAAs) have become key features for the classification and diagnosis of IIMs. To date, two different autoantibodies have been described in association with IMNM, those recognizing the signal recognition particle (SRP) and those targeting hydroxy-3-methylglutaryl-CoA reductase (HMGCR).

Autoantibodies recognizing the SRP were first identified in the 1980s [1–3]. Recently, it has been shown that the anti-SRP antibody is significantly associated with necrotizing myopathy, and the antibody is now recognized as a marker of IMNM [4].

Interstitial lung disease (ILD) is frequently associated with IIMs, especially in ASS and anti-melanoma differentiation-associated gene 5 (anti-MDA5) antibody-positive DM [5, 6]. Because ILD significantly contributes to the morbidity and mortality in patients with IIMs, it is important to clarify the clinical characteristics of ILD in such patients. Previous studies suggested that ILD was rare in IMNM patients with anti-SRP antibodies (SRP-IMNM), yet in clinical practice, ILD often occurred in conjunction with SRP-IMNM. Moreover, ILD complicating SRP-IMNM has been further substantiated in more recent reports [7–10]. However, little is known regarding the frequency, clinical characteristics, or the pattern of onset of ILD in SRP-IMNM patients. To address these questions, we reviewed the clinical data of SRP-IMNM patients intending to elucidate risk factors of ILD in these patients. Additionally, with appropriate clinical follow-up, we aimed to investigate the response to therapy of the patient subgroup, ILD in SRP-IMNM.

## Methods and materials

### Patients

All initially screened patients met either the Bohan and Peter criteria or the European Neuromuscular Centre criteria (ENMC) for IIMs between March 2010 and February 2020. All patients with IMNM were confirmed by muscle pathology. IMNM patients whose sera carried anti-SRP antibodies were selected for study. The anti-SRP antibody in the serum of patients was detected by the Euroline myositis line-blot assay of Euroimmun (Lübeck, Germany) according to the instructions of the manufacturer. Clinical and laboratory data were retrieved from their medical records, such as age, skin manifestations, muscle strength, muscle pathology, pulmonary function tests (PFTs), high-resolution computed tomography (HRCT), lung biopsy, presence of other MAAs, treatment and follow-up. If anti-SRP-positive IMNM patients were combined with other MSAs or other connective tissue diseases (CTDs), they were excluded.

This study was approved by the Ethics Institutional Review Board of the China-Japan Friendship Hospital.

### Radiologic analysis of SRP-IMNM patients

All pulmonary HRCT for the diagnosis of ILD were reviewed independently by a single experienced radiologist and a single rheumatologist. In accordance with previous reports, the HRCT patterns were classified as usual interstitial pneumonia (UIP), organizing pneumonia (OP), nonspecific interstitial pneumonia (NSIP) and lymphocytic interstitial pneumonia (LIP) [11]. The HRCT findings and disease distribution were also evaluated in detail.

### Pulmonary function tests in SRP-IMNM patients

Patients were initially divided into three groups according to the initial PFT values as referenced in previous studies: mild group (% predicted forced vital capacity [FVC] > 75% and % predicted diffusing capacity of the lung for carbon monoxide [DLCO] > 55%), moderate group (% predicted FVC from 50 to 75% or % predicted DLCO from > 35 to 55%), and severe group (% predicted FVC < 50% and % predicted DLCO < 35%) [12, 13]. Rapidly progressive ILD (RP-ILD) was defined as progressive dyspnea, progressive hypoxemia, and worsening of interstitial changes on chest radiography within 3 months from the onset of respiratory symptoms [14].

According to the consensus statement of the American Thoracic Society (ATS) on idiopathic pulmonary fibrosis, a decrease or increase of ≥ 10% in FVC was considered deterioration or improvement, respectively; either a decrease or increase of < 10% in FVC was considered stable [15].

### Statistical analysis

All analyses were completed using SPSS 21.0 and *p* < 0.05 were considered significant. Quantitative variables are reported as means and were compared using a nonparametric test. Categorical variables are reported as numbers and/or percentages and were compared using the chi-square or, when appropriate, Fisher exact test.

## Results

### Characteristics of SRP-IMNM patients

**Fig. 1:**
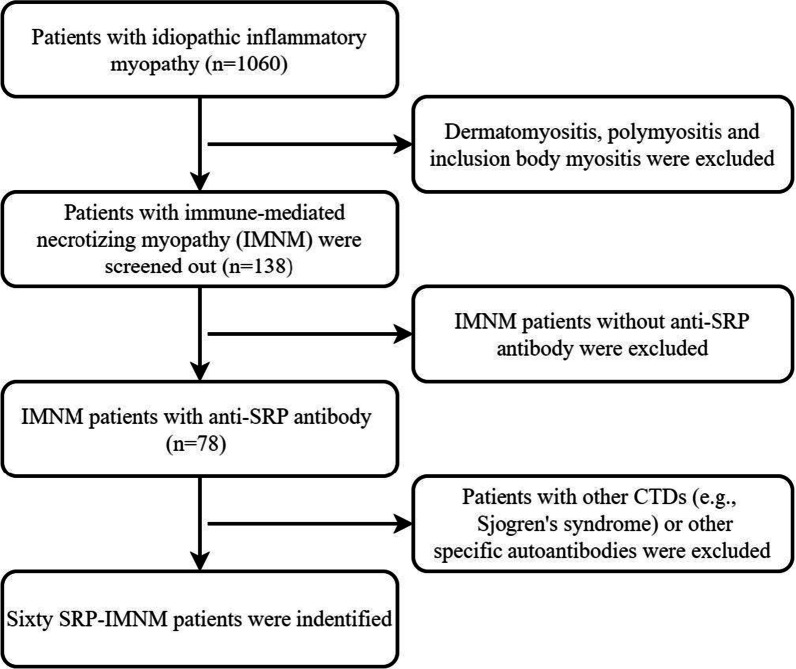
Selection flowchart

A total of 138 patients with IMNM were screened initially, included 78 patients with anti-SRP antibody. After excluding patients with other MSAs or combined with other CTDs (see Table 1), 60 IMNM patients carrying the anti-SRP antibody were identified in the study, including 45 (75.0%) females and 15 (25.0%) males. The selection process was shown in Fig. 1. The age of onset ranged from 10 to 84 years with a mean age of 44.5 ± 15.3 years. The age at onset was younger than 18 years old in 2 patients: a 12-year-old female IMNM patient with ILD and atypical skin rash; the other a 10-year-old male with IMNM.

**Table 1:** Differences between ILD and those without ILD in SRP-IMNM

Characteristic	ILD patients (n = 27)	Patients without ILD (n = 33)	*p* value
Female/male	22/5	23/10	> 0.05
Smoking history	3/27 (11.1%)	2/33 (6.1%)	> 0.05
Age (years)	48.6 ± 14.4	41.2 ± 15.4	< 0.05
Arthritis	1/27 (3.7%)	3/33 (9.1%)	> 0.05
Fever	3/27 (11.1%)	1/33 (3.0%)	> 0.05
Myalgia	11/27 (40.7%)	10/33 (30.3%)	> 0.05
Dysphagia	14/27 (51.9%)	7/33 (21.2%)	< 0.05
AST (U/L)	101.2 ± 89.5	130.8 ± 113.0	> 0.05
ALT (U/L)	126.5 ± 102.9	158.7 ± 105.8	> 0.05
CK (U/L)	2520.4 ± 2409.1	3493.4 ± 3161.7	> 0.05
LDH (U/L)	564.8 ± 316.7	705.1 ± 390.6	> 0.05
Serum ferrtin (ng/L)	162.9 ± 142.5	184.5 ± 178.4	> 0.05
ANA positive	24/27 (88.9%)	27/33 (81.8%)	> 0.05
Ro-52	13/27 (48.1%)	11/33 (33.3%)	> 0.05
SSA	4/27 (14.8%)	4/33 (12.1%)	> 0.05

LDH, lactate dehydrogenase; ALT, alanine transaminase; AST, aspartate transaminase; CK, creatine kinase

Five patients had a history of smoking, but none of them with history of dust exposures. Twenty-one (35.0%) patients suffered myalgia and 4 (6.7%) had arthralgia. Mechanic’s hands in 4/50 (8%), atypical rash in 5/60 (8.3%) and Raynaud's phenomenon occurred in 2/50 patients (4%).

Cardiac involvement was observed in 6/60 patients (10.0%), presenting as cardiac troponin I (cTnI) elevation, myocardial ischemia, arrhythmia, or myocardial MRI that demonstrated myocardial fibrosis. Five (9.1%) patients developed mild pulmonary arterial hypertension (PAH). Cancers were observed in 5 patients (8.3%).

Fifty-one (85.0%) patients were ANA-positive, with a titer ranging from 1:80 to 1:640, and the most common pattern of ANA was cytoplasmic granules. Patients testing positive for other MAAs included 24 (40.0%) patients with Ro-52, 8 (13.3%) patients carried the SSA antibody.

### Radiologic analysis of ILD in SRP-IMNM

Fifty patients were evaluated with HRCT and and 10 patients by X-ray. In total, 27 (45.0%) patients were diagnosed with ILD on the basis of lung images or pulmonary histopathology. The radiologic patterns of ILD included NSIP in 17 patients (63.0%), OP in 9 (33.3%), and one patient with LIP (3.7%).

**Fig. 2:**
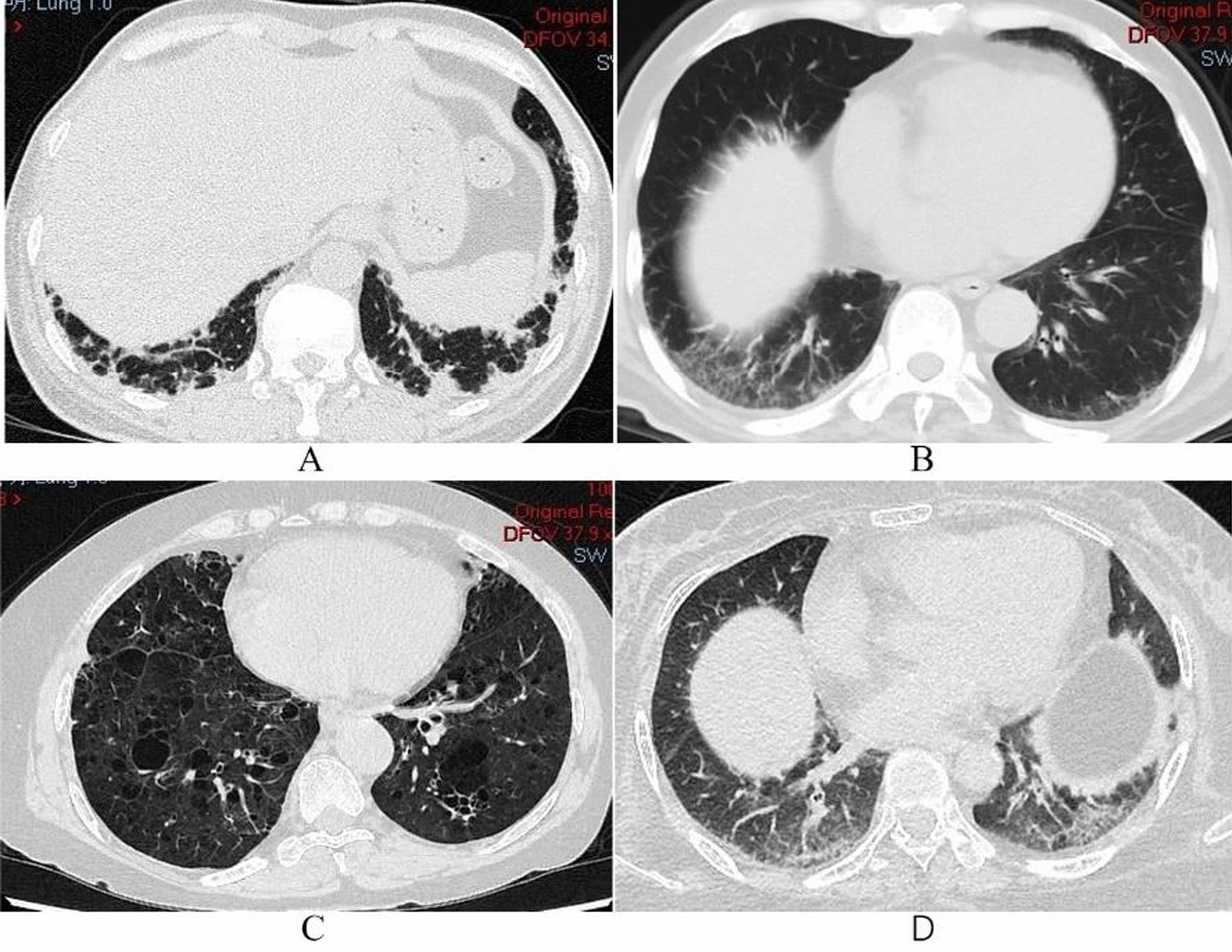
Abnormal opacities of HRCT from different SRP-IMNM patients. **A** 45-year-old woman with OP; **B** 54-year-old man with NSIP; **C** 55-year-old woman with LIP; **D** 45-year-old woman with NSIP

In most cases, abnormal opacities were predominantly distributed in the lower lobes and peribronchovascular sites. Ground glass opacity (GGO) was identified in 10 (37.0%) patients, 17 (63.0%) showed reticulation, 10 (37.0%) had patchy shadows, 6 (22.2%) had interlobular septal thickening (IST), there were 5 (18.5%) with pleural thickening, and consolidation was found in 9 (33.3%) cases. In contrast, traction bronchiectasis (TB, 7.4%) and cyst (3.7%) were infrequently seen. Representative HRCT images are shown in Fig. 2.

### Severity of ILD in SRP-IMNM

Of the 27 SRP-IMNM patients with ILD, most were asymptomatic at the time of disease onset with slow development without respiratory symptoms; only 12 (44.4%) patients experienced dry cough and breathing difficulty with activity.

The PFTs were available for 18 patients: 6 (33.3%) met criteria for inclusion in the mild group, 10 (55.6%) comprised the moderate group, and two (11.1%) was in the severe group based on PFTs. None of the patients progressed to RPILD. In addition, all patients had FEV1/FVC ≥ 70%.

### Comparison of SRP-IMNM patients with and without ILD

Comparing the two groups, the average age of onset among SRP-IMNM patients with ILD was significantly older than those without ILD (48.6 ± 14.4 years vs. 41.2 ± 15.4 years, *p* < 0.05) (see Table 1). The frequency of dysphagia in the ILD group was higher than the group without ILD (*p* < 0.05). The frequency of smoking history, skin rashes, arthritis, fever, myalgia and mechanic’s hands in the ILD group was not significantly different from the group without ILD (*p* > 0.05). Serum creatine kinase (CK), lactate dehydrogenase (LDH), as well as ALT and AST levels in ILD patients were not significantly different from those without ILD (*p* > 0.05). Regarding MAAs, the frequency of ANA, SSA and Ro-52 antibody were somewhat higher in the ILD group than patients without ILD, but the differences did not reach statistical significance (all *p* values > 0.05).

### Follow-up of ILD in SRP-IMNM patients

**Fig. 3:**
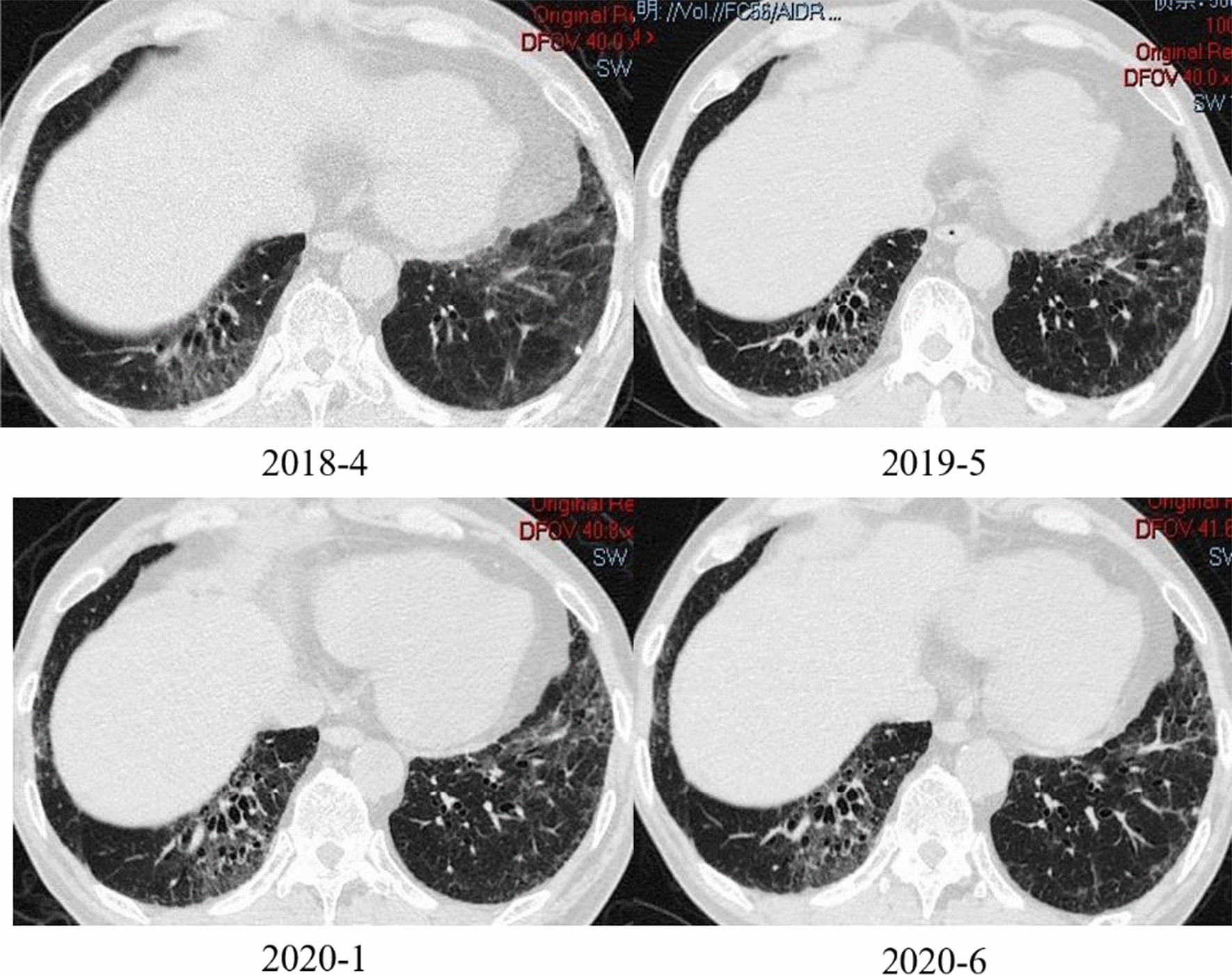
A 55-year-old male patient's lung CT, almost unchanged in 26 months

**Fig. 4:**
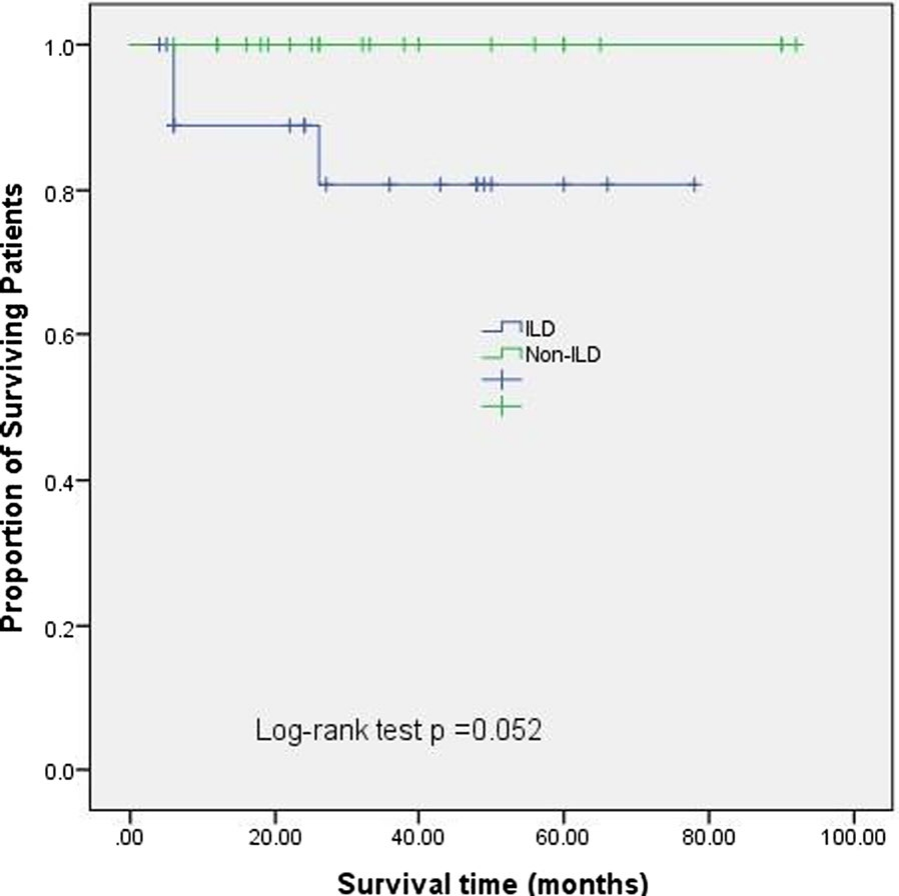
Kaplan–Meier survival curves for patients with and without ILD

Of the 60 patients, three patients with ILD died during follow-up; one patient died of pulmonary embolism and infection, one patient died of severe pulmonary infection, and another patient died due to the deterioration of ILD. Nine of the ILD patients were followed with HRCT and PFTs for a median of 23 months (range, 6–78 months) (Table 2). One patient received only glucocorticoids whereas the remaining 8 patients received a combination of immunosuppressive agents such as azathioprine (AZA), tacrolimus (TAC), cyclophosphamide (CYC), intravenous immunoglobulin (IVIG), and Tocilizumab (TCZ). Eight patients responded well to glucocorticoid and/or immunosuppressive agents with improvement in myositis. Moreover, patients 2 and 8 experienced restoration of normal muscle strength and CK levels. In accordance with performance standards for PFTs, 8 (88.9%) patients remained stable, and worsened in one. On assessment of HRCT, 6 (66.7%) patients remained stable (see Fig. 3), but progression of ILD occurred in 3 patients with the appearance of a new abnormal shadow. Kaplan–Meier showed that the survival rate of ILD group was lower than that of non ILD group, but the results were not statistically different (Fig. 4).

**Table 2:** Changes in ILD patients at admission and the last follow-up

Patients, gender, age	Admission						Last visit						
	MMT	CK	FVC (%)	FEV1/FVC (%)	DLCO (%)	HRCT	MMT	CK	FVC (%)	FEV1/FVC (%)	DLCO (%)	HRCT	Fellow-up (months)
P1, F, 48	65	7203	71	81	63	GGO	78	1180	59	82	58	GGO, reticulation	43
P2, F, 66	72	1436	99	71	88	Reticulation	80	25	114	72	74	Reticulation	6
P3, F, 41	68	4113	82	85	82	IST	70	51	84	100	66	IST	40
P4, F, 51	70	2748	71	86	58	Reticulation	72	171	69	88	68	Reticulation	78
P5, F, 24	56	6367	69	83	70	Reticulation, IST	75	43	77	100	49	Reticulation, IST	23
P6, M, 55	55	1620	83	90	64	GGO, consolidation	63	661	83	93	65	GGO, consolidation	9
P7, F, 50	80	1176	90	85	50	Reticulation	80	1702	97	87	56	Reticulation, IST, TB,	48
P8, F, 52	75	535	70	83	58	Reticulation, IST	80	34	65	83	55	Consolidation, Patchy shadows	6
P9, M, 54	45	3196	57	96	71	GGO, patchy shadows, PT	44	2459	59	81	60	GGO, PT, patchy shadows	7

GGO, Ground glass opacity; IST, interlobular septal thickening; TB, traction bronchiectasis; MMT, manual muscle test; PT, pleural thickening

## Discussion

The present study investigated the clinical and radiological features and prognosis in SRP-IMNM patients with ILD. About half of SRP-IMNM patients were diagnosed with ILD. On HRCT, greater than half of the patients showed reticulation, followed by a pattern of GGO and patchy shadows. NSIP was the principal radiological pattern in ILD of SRP-IMNM patients. The severity of ILD in most patients was classified as mild to moderate as judged by PFTs and remained stable in most patients.

SRP is a complex consisting of six polypeptides and a 7S RNA molecule that is important for the translocation of newly formed proteins to the endoplasmic reticulum [2]. The hallmark feature of patients with anti-SRP antibodies is the presence of proximal muscle weakness, myofiber necrosis with minimal inflammatory cell infiltrate on muscle biopsy. The clinical picture of muscle weakness and characteristic pathologic changes of IMNM were present in all patients in our cohort.

Extra-muscular manifestations such as skin, lung and heart involvement may also be seen in SRP-IMNM patients. Pinal-Fernandez et al. reviewed the characteristics of patients with IMNM, and ILD occurred in only 10–20% of SRP-IMNM patients and fewer than 5% of those with HMGCR-IMNM [4]. Similarly, in a study of 100 Japanese IIM patients with anti-SRP antibodies, 13 (13%) patients had ILD [7]. In a literature review of SRP-IMNM, including 9 case series with a total of 239 patients, only 41 (17%) patients had ILD [8]. In sharp contrast, nearly half of our SRP-IMNM patients had documented ILD. The prevalence of ILD was higher in our study, but it still needed to be confirmed by a larger cohort in the future.

In our cohort, analysis of HRCT revealed that the abnormalities were predominantly distributed in the lung bases, and greater than half of the SRP-IMNM patients had reticulation. Additionally, GGO, patchy shadows, and IST were commonly identified by HRCT. Furthermore, the HRCT patterns of ILD in SRP-IMNM patients were most commonly NSIP, followed by OP; LIP and UIP were rarely identified. In 2010, Wantke et al. reported a 46-year-old female SRP-IMNM patient with OP, yet the patient was completely stable and lung function even improved. The HRCT showed no sign of active OP but rather, diffuse fibrosis especially in the middle lobe [9]. In 2015, Suzuki described the clinical characteristics of 100 patients with anti-SRP antibodies. Chest CT revealed ILD in 13 patients, all of whom had NSIP, and their respiratory symptoms were generally mild [7]. Kusumoto et al. described NSIP in a 72-year-old male SRP-IMNM patient: chest CT revealed GGO and reticular shadows with a peripheral, predominantly lower lobe distribution. Treatment with prednisolone and immunosuppressive agents resulted in normalization of CK, mild muscle weakness, and CT and PFT showed no further progression of ILD [8].

Because the vast majority of our patients did not undergo bronchoscopy or surgical lung biopsy, confirmation of HRCT-based subtyping of ILD was lacking and may not have been entirely accurate. In our cohort, only a single female child with ILD was found, an extremely rare occurrence. Standard chest X-ray or plain CT scan may not be adequate to diagnose ILD, so screening for it should include HRCT.

Although ILD is frequently present in SRP-IMNM patients, it is typically not severe, as only a small number of patients in our series noted dyspnea with exercise. With objective PFT measurements, about 90% of patients with ILD were classified as mild or moderate. In contradistinction, for patients with ASS and anti-MDA5 antibody positive DM, the severity of ILD is often moderate to severe, and some progress to RP-ILD. Another study observed that subclinical lung involvement was not rare in patients with IMM associated with positive anti-NXP2 antibody, with various radiological patterns and a significant lung function defect [16]. Patients with SRP-IMNM may not only develop proximal muscle weakness, but diaphragmatic muscles can also be involved, resulting in decreased lung function. This may lead to restrictive ventilation dysfunction. In addition, our study showed that ILD group had a higher frequency of dysphagia, which was easy to lead to gastroesophageal reflux. These factors may be involved in the occurrence and development of ILD [17].

The patients with ILD in our cohort were middle aged, which suggests that screening for ILD with HRCT should be performed regularly in this age group of SRP-IMNM patients.

The prognosis for SRP-IMNM patients is generally good, as most patients can anticipate long term survival. Comparing the patients with and without ILD, the ILD group had a relatively higher death rate. However, only one patient with ILD died as a direct consequence of this complication whereas the 2 other deaths in this group resulted from a lung infection and a pulmonary embolism.

Despite the severity of the ILD being classified as mild to moderate, it did not improve with the recovery of muscle strength, ILD remained relatively stable. This outcome may be a function of relatively short follow-up.

Two of our patients did have severe ILD. Similarly, Qureshi et al. presented a 40-year-old female who presented with acute hypoxic respiratory failure secondary to SRP-IMNM associated ILD. After struggling through a complicated 6-month hospitalization, the patient underwent successful double lung transplantation and was eventually discharged on room air [10].

The present study has several limitations. First, it was a retrospective, single-center study with a relatively small sample size. Second, by the nature of a retrospective, single center (specializing in respiratory diseases) study, selection bias might have played a role. Third, the gold standard assay for the detection of MSAs is the immunoprecipitation assay (IP), but MSAs were detected by line blot assay in our study, this may lead to false positive or false negative.

In conclusion, the present study demonstrated that the complication of ILD in SRP-IMNM patients occurred appreciably more frequently than in previously published reports, especially in older middle-aged patients. However, the severity was limited to only mild or moderate in most patients and remained stable throughout the follow-up period.

## Data Availability

The original contributions generated for the study are included in the article, further inquiries can be directed to the corresponding author.
